# Certain Powers and Properties of Water

**Published:** 1873-10

**Authors:** P. H. Hayes


### Certain Powers and Properties of Water.

P. H. HAYES, M. D.

Have you read that fanciful story of Edmund About, entitled "The Man With the Broken Ear," and do you recall how the colonel, sentenced to die, was made the subject of a grand experiment. Instead of being executed, his sentence was commuted to being literally dried up as dry as a mummy, and then the problem was to see whether he would not revive, after being kept a half century, by slowly and cautiously supplying the amount of water withdrawn by the drying process.—But would you suppose, if this colonel weighed a hundred and fifty pounds, that the drying process, when perfectly complete, would actually take away a hundred and twenty pounds, and leave the dried up man only thirty pounds? Yet such is the fact, that four-fifths of the body is water, and this water is so intimately combined with every part and substance of the body that there could be no life, no circulation, no sensation, no thought, no motion without it. Thus the brain is more than four-fifths water, and from the amount of this fluid the organ is so soft and delicate as to require the hardest, horny casement for its protection. Moreover those nerves which minister to the sense of sight, are required to be so extremely delicate in order that they perceive the light at all, that they are half suspended in the fluids of the eye. The same is even more true of the nerves of the ear which fairly float in the fluid of the semi-circular canals. How very intimately, then, is water associated and bound up with the organization and the mystery of life in its most subtle and mysterious manifestations.

Of the blood seven hundred and ninety parts in a thousand are water, and here we learn that water is the only proper solvent for our food, and the only medium of conveyance for all the elements of the blood which are to nourish and build up the body. Moreover, water is the medium of solution for all the waste of the body, the old effete and worn-out elements which must pass out of the body by the lungs—the breath is full of vapor—the liver, the skin, &c.

We may gather a hint of the intimate relation of water to life in the urgency of thirst, the torments of which are intolerable, with which the intensest degree of hunger is not for a moment to be compared.

It is but a step to infer that an element so instantly essential to life might be of the utmost importance in the *renewal* of life in all states of disease. And so experience has proven, that in acute diseases always attended by fever, there is no one agent so efficient to remove it as water.

It is a wonderful law of the human organism that all its powers and actions can only go on healthfully at a temperature of ninety-eight Fahrenheit. Fever raises the body above this standard, and then digestion and nutritition are suspended and disturbance and exhaustion follow. Water drank freely and used in tepid sponging of the surface lowers the bodily heat until the normal standard is reached, and then digestion and nutrition return, and life goes on as before.—The author has seen severe fevers broken almost alone by water-drinking, taking a little and often, and persevering until perspiration came on.

In chronic diseases, baths of a moderate temperature, with hand-rubbing to promote reaction, increase the vigor of the skin, and cause the blood to be purified more perfectly through the millions of glands which the skin contains. In health, the skin carries off by insensible perspiration about one-third of all the waste and worn out elements of the body. If the skin were to lose this power utterly for a single day, the result would be death, just as five minutes total suspension of breathing would also be fatal, for in either case the body is poisoned by retaining the morbid matters which should have passed out by these organs. It is a curious law that nature in curing diseases throws off the disease from more vital to less vital parts, as she works from within toward the surface. Thus in scarlet fever, measles and small-pox, erysipelas, &c., nature expels the virus from within, outward upon the skin, where it does far less harm, and common observation teaches the great danger of repelling the eruption, or causing it to "strike in," for then vital organs are liable to be attacked, and death follow.—Even in gout and rheumatism, diseases characterised by humors in the blood, as their name indicates, the disease may be driven from the joints to the heart, lungs and stomach, and even to the brain. But while nature has strength, she will keep the virus which is the cause of these diseases in the joints, the outposts of the body, that vital organs may not be harmed. It is therefore easy to see that all those forms of bathing which invite the blood into the skin and the extremities and increase vital action and elimination of waste and humors here, does by so much exalt and renew the life forces, and is a mode of working with nature, or *like* nature in the cure of disease.

Again the skin is, so to speak, a vast expanse of nerves, it being impossible to put down so much as the point of a cambric needle without its being felt. All outside impressions made upon this sheet of nerves have their internal counterparts. Thus when we suddenly enter the cool bath, or have the water poured upon the body, we instantly catch our breath involuntarily, the impression upon the skin being, so to speak, instantly *felt* by the lungs. Thus by a wise application of water to the skin, internal congestions and obstructions may be removed, the stimulus felt by the skin being reciprocated by internal organs. Thus according to the temperature, water may be made to act as a tonic or stimulant, a sedative or an anodyne. The pleasant glow and sense of new life which follows the wise use of the bath is familiar to all habitual bathers.

Again, regular bathing greatly accelerates the changes of waste and repair which are constantly going on in the body. The medium by which these changes take place is the blood. We eat, and say we are nourished by our bread and butter, by our beef steak, but strictly speaking, we are not nourished by these things, but by the blood the digestive organs can make from them. The blood is the "life of the flesh," and so it conveys to each part what it needs for its own reconstruction, receives in turn the elements that have already served their use in the body. About one-seventh part of the weight of the body is blood. Thus a man of one hundred and forty pounds, has about twenty pounds of blood. But every day of his life about one-half pound of this blood enters into the structure of his body, and another half pound is eliminated from the blood by the lungs, liver, skin, &c., in the worn out particles of the body. Now it is easy to see that if by any process these changes are greatly increased, and if at the same time new and better blood is made by a more healthy digestion, then we shall get a better and better blood, and from this will a more and more healthy body be built up, and thus finally the entire system undergo a healthy reconstruction.

In all the processes of bathing, we speak of the skin as being *wet* by the water, yet strictly speaking the skin cannot be wet with water at all. If we examine closely, we shall see that the water only touches the skin, and stands upon it very much as it does upon a duck's back. In all natural states of the skin it is polished upon its surface by the sebum or oil from the sebaceous glands of the skin, and it is only when this is washed out by soap that the skin can be wet with water, and it then becomes soft and spongy as often may be seen in the hands of washerwomen.—Strangely enough, although you cannot wet the skin with water, yet steam will wet the skin and be absorbed by it very rapidly, even to the extent of a pound or more during the vapor bath, and the headache and labored action of the heart sometimes felt in these baths is no doubt due in part to this sudden loading of the circulation.

Though the skin cannot be wet with water, oil will wet it instantly, penetrating to close contact with the absorbents, and without doubt entering the circulation. The oil bath is particularly useful in all dry, half-dead states of the skin, as it gives the laxity and softness necessary to health. This quality of oil to penetrate pores or tubes where oil already exists, may be turned to good account in killing insects in out of the way places, for although you cannot drown them in water, they will instantly drown in oil. This is owing to the fact that the minute stigmata or bronchial tubes all along the sides of the body of the insects are coated with oil, and water will not enter, but oil penetrates quickly, stops the breathing, and extinguishes the insect. Glycerine has this same quality of penetrating parts already oiled, and may be used as well as oil, though not so agreeable, to destroy an insect which may have penetrated into the ear, where most speedy destruction can thus be accomplished, and then the insect be withdrawn entire by a delicate pair of forceps.

